# Structural analysis of cross-linked poly(vinyl alcohol) using high-field DNP-NMR†

**DOI:** 10.1039/d0ra00399a

**Published:** 2020-02-24

**Authors:** Taiji Kanda, Mayuka Kitawaki, Toshiaki Arata, Yoh Matsuki, Toshimichi Fujiwara

**Affiliations:** Mitsubishi Chemical Corporation 2-13-1, Muroyama Ibaraki Osaka Japan kanda.taiji.mp@m-chemical.co.jp; Institute for Protein Research, Osaka University 3-2, Yamadaoka Suita Osaka Japan

## Abstract

Poly(vinyl alcohol) (PVOH) is a water-soluble synthetic polymer, widely used in materials for functional films and moldings, fiber fabric sizing agents, paper coating resins, and adhesives. PVOH is mainly applied in the form of an aqueous solution, yet after its application, insolubility (water resistance) is required. To achieve this, additives are introduced. These additives used with PVOH are cross-linking agents which react with the hydroxyl groups and modified functional groups in PVOH. Because of the poor reactivity of unmodified PVOH, it does not react with cross-linking agents that have functional reactive groups. Therefore, modified PVOH that reacts with a cross-linking agent more successfully is required. These chemical bonding sites are so low in abundance that it is difficult to characterize the cross-linking structure. Solid-state ^13^C NMR is a powerful technique that can be used for the structural analysis of a polymer material. However, its sensitivity is low, hence it is difficult to determine crosslinking in a polymer, as it makes up only a small proportion of the product. Therefore, solid-state ^13^C NMR sensitivity can be enhanced by high-field dynamic nuclear polarization (DNP) using strong electron polarization. In this study, the reaction of acetoacetylated PVOH with a cross-linking agent, adipic dihydrazide, was analyzed. This crosslinked PVOH is the most popular vinyl alcohol polymer on the commercial market. The sensitivity enhanced ^13^C NMR spectra reveal that the carbonyl of the acetoacetyl group of PVOH crosslinks with adipic hydrazide by forming an imine bond (>C=N–) this study also shows that the product has only seven crosslinking sites per molecular chain with a polymerization degree of 1000 and is water resistant.

## Abstract 2

Poly(vinyl alcohol) (PVOH) is a water-soluble synthetic polymer, widely used in materials for functional films and moldings, fiber fabric sizing agents, paper coating resins, and adhesives.

## Graphical abstract

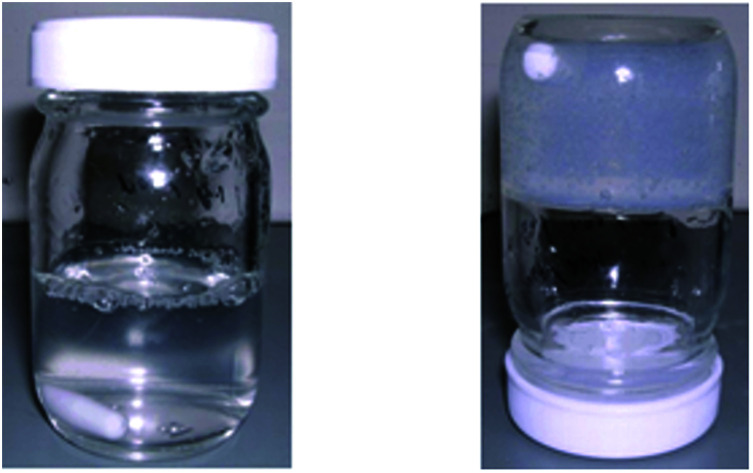

## Introduction

Vinyl alcohol polymers are synthetic polymers, the most representative examples of which are poly(vinyl alcohol) (PVOH) and ethylene vinyl alcohol copolymer. Vinyl alcohol polymers are widely used in applications such as optical polarizing films,^1,2^ food packaging films,^3^ as dispersing agents,^4,5^ fibers,^6^ overcoating agents for paper,^7^ pharmaceutical additives,^8^ and various other additives. As these materials are used in the solid state by consumers, it is necessary for quality and manufacturing purposes to improve the physical properties of these materials, to have control over their design, in terms of characteristics such as hydrogen bonding and conformation, and understand their properties.

One of the main applications of PVOH is in coatings. PVOH can be dissolved in water and coated onto a backing material, such as paper or a film. After application it is made insoluble, to achieve durability.

**Fig. 1:**
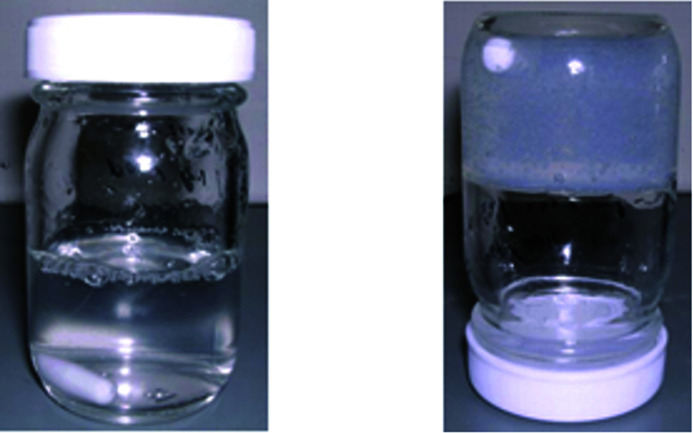
PVOH: immediately after adding a cross-linking agent to an aqueous solution (left) and after several hours (right).

Insolubility can be achieved by adding a cross-linking agent that reacts with the hydroxyl groups of the PVOH. To date, various cross-linking agents such as dialdehydes (glyoxal, glutaraldehyde), dicarboxylic acids (malonic acid, succinic acid, citric acid),^9^ boronic acid, borates^10^ and borax have been developed. However, the cross-linking speed and the insolubility of the cross-linked product were not sufficient. To solve this problem, modified PVOH has been introduced with modified functional groups that are highly reactive, with which a cross-linking agent can react. PVOH with an acetoacetyl functional group is a highly reactive form of PVOH. Adipic dihydrazide (ADH) has been used as a cross-linking agent for acetoacetylated PVOH. When an aqueous solution of acetoacetylated PVOH and ADH are mixed, a highly viscous aqueous solution forms immediately, as shown in Fig. 1. After a few minutes it forms a gel—it is then no longer soluble in water. This change occurs rapidly and the water resistance performance changes dramatically. This behaviour can be detected in the dynamic viscoelasticity of the material and the measurement of any undissolved residues. However, the mechanism of this reaction is still unknown. One of the reasons for this is that only a small portion of the structure reacts, hence it is very difficult to analyze the structure.

Solid-state ^13^C nuclear magnetic resonance (NMR) provides information on many structures and molecular motions of polymers, including PVOH in the solid state. However, NMR has the disadvantage that its sensitivity is much lower than that of some other analytical methods. At equilibrium, the difference in the populations of the upper and lower states is only 1 in 200 000 spins at 16.4 T [^1^H Larmor frequency: 700 MHz] at room temperature. Therefore, the lower the transition energy in NMR, the lower the sensitivity. Moreover, the low sensitivity of ^13^C-NMR is not only due to the energy being lower than that of other spectroscopy techniques, but also due to the low natural abundance of ^13^C in organic compounds.

To overcome this rather low sensitivity of NMR, in solid-state NMR, the method of cross-polarization/magic angle spinning (CP/MAS) in which the magnetization of the ^1^H is transferred to the magnetization of ^13^C, to improve the sensitivity by up to four times, has been widely used. However, there appears to be no way, as yet, to increase the number of acquisitions to assign the microstructure. Now, if the magnetization can be transferred from an electron spin with a high magnetogyric ratio to a nuclear spin with a low magnetogyric ratio, a significant improvement in the sensitivity can be achieved (660 times better for ^1^H and 2640 times better for ^13^C).

Dynamic nuclear polarization-NMR (DNP-NMR) involves the transfer of magnetization from electron to nuclear spins, resulting in a significant enhancement in the sensitivity of the resonance line. There are four main mechanisms applicable in DNP, including the Overhauser effect, the solid effect, the cross effect, and thermal mixing. Examples of the successful use of DNP-NMR for synthetic polymers include the elucidation of the interfacial structures of polystyrene and polycarbonate blends,^11,12^ and as a probe, to increase the sensitivity of the interfacial part by embedding a polarizable component in a polymer. For the quantitative analysis of the chemical composition, –(CX_2_)_*n*_– [X = ^1^H, ^2^D, ^19^F] doped with TEMPO (2,2,6,6-tetramethypiperidiny-1-oxyl free radical) has been investigated.^13^ Furthermore, the cross-linked portion of a polyimide determined using DNP-NMR has been reported, where improved sensitivity to the portion to be observed was achieved.^14^ There are also reports on the use of DNP-NMR to analyze polyethylene, poly(ethylene oxide), polylactide, and poly(methyl methacrylate), prepared by film casting or glass formation, and the rates of the polarization transfer were determined.^15^ Furthermore, recently the structures of cross-linked polystyrene^16^ and collagen^17^ were detected using DNP-NMR, made possible by improving the high field. However, the analysis of synthetic polymers using DNP-NMR is currently less common than that of proteins and low molecular weight compounds (^13^C urea, for example), which are commonly used as target compounds to measure the polarization effect. In particular, the cross-linked structure in polymers is not detected because it constitutes only a small proportion of the overall polymer structure. There is currently no analytical method that is good enough to analyze this small proportion in insoluble polymers. Investigations into cross-linked structures would reveal not only how they react, but also provide valuable information on important effects on polymer processes and on productivity, such as processing and manufacturing, upon control of the reaction rate. Therefore, we studied the structure of PVOH, in which the reactive acetoacetyl groups in PVOH were reacted with a cross-linking agent, and monitored it using high sensitivity ^13^C NMR *via* DNP.

## Experimental

### Materials and methods

**Fig. 2:**
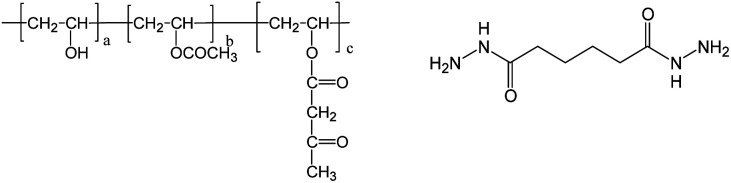
The structures of acetoacetylated PVOH (left) and ADH (right).

Acetoacetylated PVOH, in which the PVOH has a degree of polymerization of 2400, 98 mol% saponification value, and 4 mol% acetoacetylated content (Mitsubishi Chemical Corporation, Tokyo, Japan) was selected for use. ADH (Otsuka Chemical Corporation, Tokyo, Japan) was used as the cross-linking agent. The structures of acetoacetylated PVOH and ADH are shown in Fig. 2.

At room temperature, 25 g of a 5% acetoacetylated PVOH aqueous solution and 1.25 g of a 10% ADH aqueous solution were mixed. The resulting mixture was immediately cast onto a polyethylene terephthalate film (thickness 100 μm), then dried for two weeks at room temperature. The resulting film was not soluble in water, it only swelled. The film was freeze crushed and then 19 mg of the crushed powder and a 20 mM solution (20 μl) of TOTAPOL (DyNuPol, Inc., Massachusetts, USA,^18,19^ including biradicals as a polarizing agent) were dissolved in 12.3 μl of a glycerol matrix (solvent d_8_-glycerol/D_2_O/H_2_O = 6/3/1 w/w/w), then filled into a 10 μl PTFE sample holder. Measurements were conducted on a high-field DNP-NMR instrument at Osaka University.^20^

**Fig. 3:**
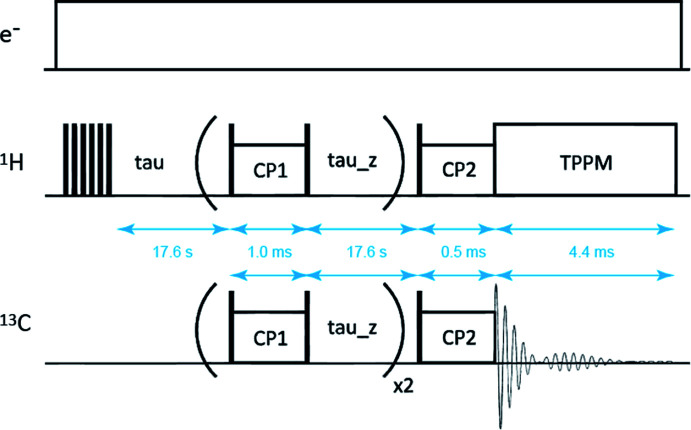
Pulse sequence used for DNP/CP/MAS measurements. The conditions are as follows. Gyrotron: FU CW GOI apparatus was used, at an electron acceleration voltage of 18 kV and a beam current of 270 mA. NMR: a JEOL ECA 700 spectrometer was used with a 3.2 mm HC probe and a TPPM decoupling sequence.^22^

Samples were cooled to 100 K in a 3.2 mm Si_3_N_4_ rotor and spun at 8000 Hz under MAS. ^13^C NMR spectra were recorded using a JEOL resonance 700 NMR spectrometer operating at *B*_0_ = 16.4 T (700 MHz ^1^H and 175 MHz ^13^C Larmor frequencies) with a gyrotron operating at 462 GHz. Spectra were obtained from the magnetization transfer in ^1^H spins to the ^13^C spins *via* a CP process during excited electron spins. The CP process uses multiple CP methods.^21^ The pulse sequence can quantitatively capture signals from different functional groups that arise from polarization buildup times (*T*_CH_) and rotational spin–lattice relaxation times (*T*_1ρH_). The pulse sequence used is shown in Fig. 3, where CP1 and CP2 were set to 1 and 0.5 ms, respectively. Free induction decay signals were obtained during a 4.4 ms acquisition time, followed by a 0.5 s repetition time.

## Results

**Fig. 4:**
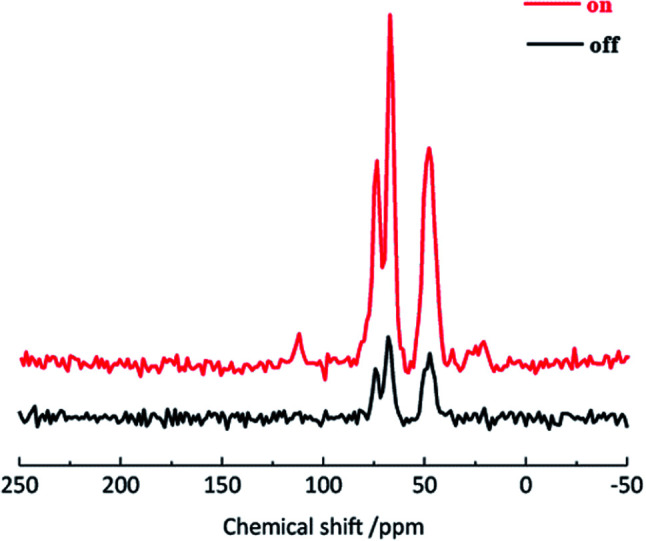
^13^C DNP-CPMAS spectra of cross-linked PVOH in the presence and absence of microwave irradiation.

^13^C DNP-CPMAS spectra of cross-linked PVOH in the presence and absence of microwave irradiation, for two acquisitions, are shown in Fig. 4 to compare the peak intensities.

After irradiation, improved sensitivity of the resonance line of the PVOH main chain was achieved, and improved sensitivity was confirmed, for the first time, in the high-field DNP-NMR analysis of PVOH. The ratios of the height of the resonance lines, the enhancement factor (*ε*_DNP_), post microwave irradiation, was 3.8 for the PVOH main chain (45 ppm) and 4.1 for glycerol (70 ppm). Because the *ε*_DNP_ value for PVOH is similar to that of glycerol, it was thought that the polarization effect was due to the glycerol matrix.

**Fig. 5:**
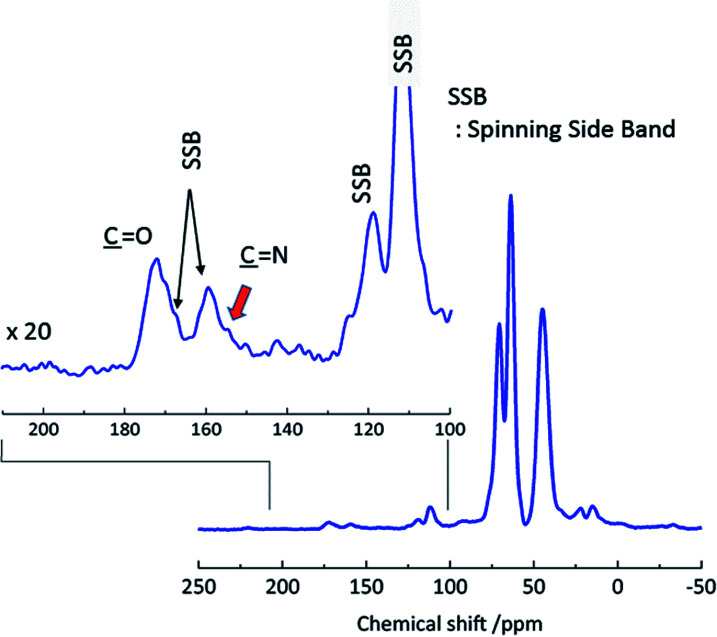
^13^C DNP-CPMAS spectra of cross-linked PVOH under microwave irradiation. Number of acquisitions: 430.

Fig. 5 shows the ^13^C DNP-CPMAS spectra of cross-linked PVOH under microwave irradiation conditions. The inset shows a 20× magnification, between 100 and 200 ppm. In this spectrum, all resonance lines were detected, specifically/including those of the PVOH main chain, the modified part, and the methylene of ADH. A new resonance line was observed at 156 ppm.

**Fig. 6:**
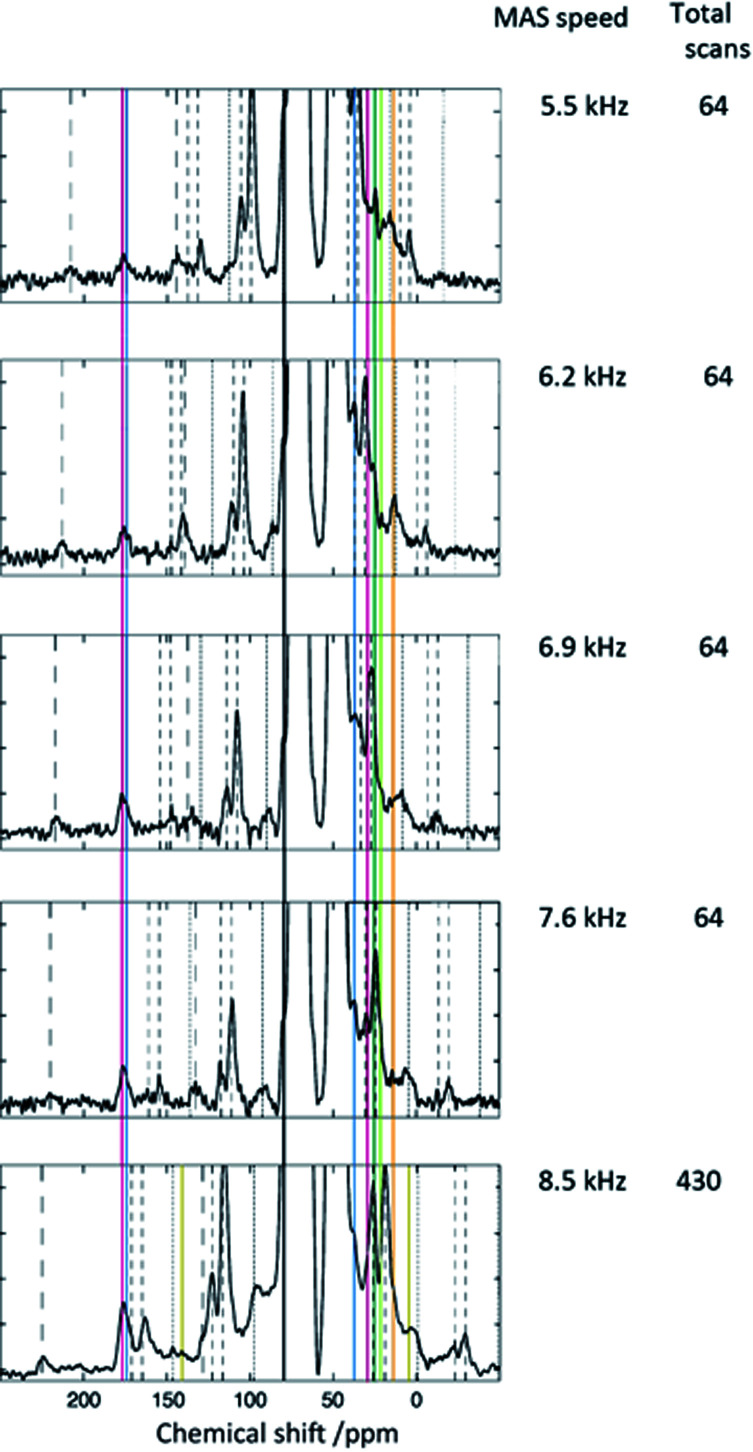
^13^C DNP-CPMAS spectra obtained as a function of the changing MAS frequency.

**Fig. 7:**
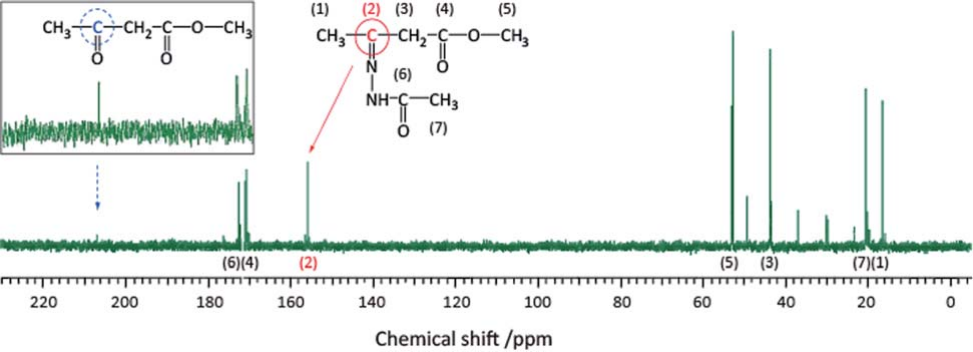
Solution ^13^C NMR spectrum of the reaction of methyl acetoacetate and acetohydrazide (inset: just after the mixing of equal amounts of methyl acetoacetate and acetohydrazide).

**Fig. 8:**
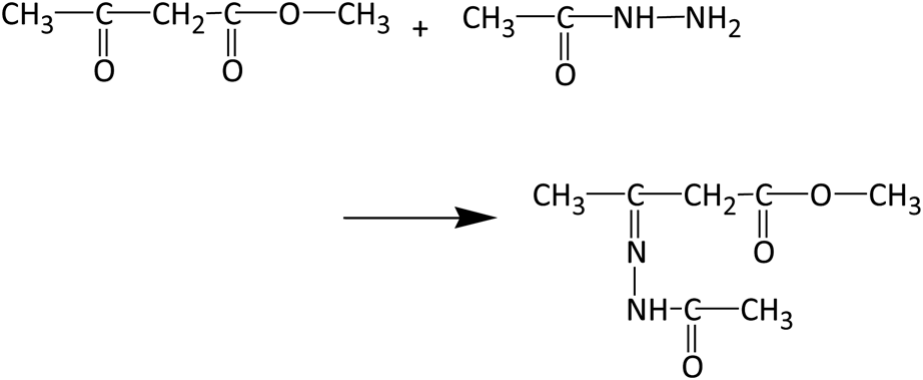
Reaction of methyl acetoacetate and acetohydrazide.

The spectra in Fig. 6 were obtained at a MAS frequency from 5.5 to 8.5 kHz. The spinning sidebands are indicated by the dashed lines. The peaks were observed in the region not overlapping the spinning sidebands, *i.e.*, in the resonance lines indicated by the colored solid lines. A resonance line at 156 ppm (indicated by the magenta line) was also detected. Although this peak was rather unclear, it was present in most of the spectra, particularly in the spectra with a large number of total scans. An experiment with a low molecular weight compound was carried out to determine the chemical structure for the peak at 156 ppm. Methyl acetoacetate, which has only the reactive part of acetoacetylated PVOH, and acetohydrazide, which has the reactive part of ADH, were mixed, and the product analyzed using solution NMR after 1 h. The reaction between methyl acetoacetate and acetohydrazide in solution is described in more detail in the ESI.† Methyl acetoacetate and acetohydrazide were mixed in equivalent amounts in water at room temperature. The structure of the product was identified using solution ^13^C NMR. The spectrum is shown in Fig. 7, where the resonance peak at 156 ppm can be observed. On the other hand, the resonance peak at 207 ppm for the ketone in methyl acetoacetate almost disappears. The reaction is shown in Fig. 8.

Because the chemical shift of the resonance line in the model reaction is similar to that in the cross-linked structure of the polymer, it should arise from the same chemical structure.

## Discussion

Solution ^13^C NMR of a mixture of methyl acetoacetate and acetohydrazide was compared with that of the reactants alone. A peak disappeared at 207 ppm and a peak appeared at 156 ppm (Fig. 7). The peak at 207 ppm represents the carbonyl group of the methyl acetoacetate. The peak at 156 ppm in the spectrum can be attributed to an imine, formed after the reaction of methyl acetoacetate and acetohydrazide. Immediately upon mixing the acetoacetate and acetohydrazide, the carbonyl group (C=O) of the methyl acetoacetate reacts with the hydrazide group (NH_2_) of the acetohydrazide, forming an imine bond. Thereafter, a pyrazolone ring is formed. This has been confirmed with similar compounds.^23,24^ However, because fewer reactions occur in the polymer form than in the smaller model compound form, it is likely that the reaction rate of the polymer and hydrazide is slow.

**Fig. 9:**
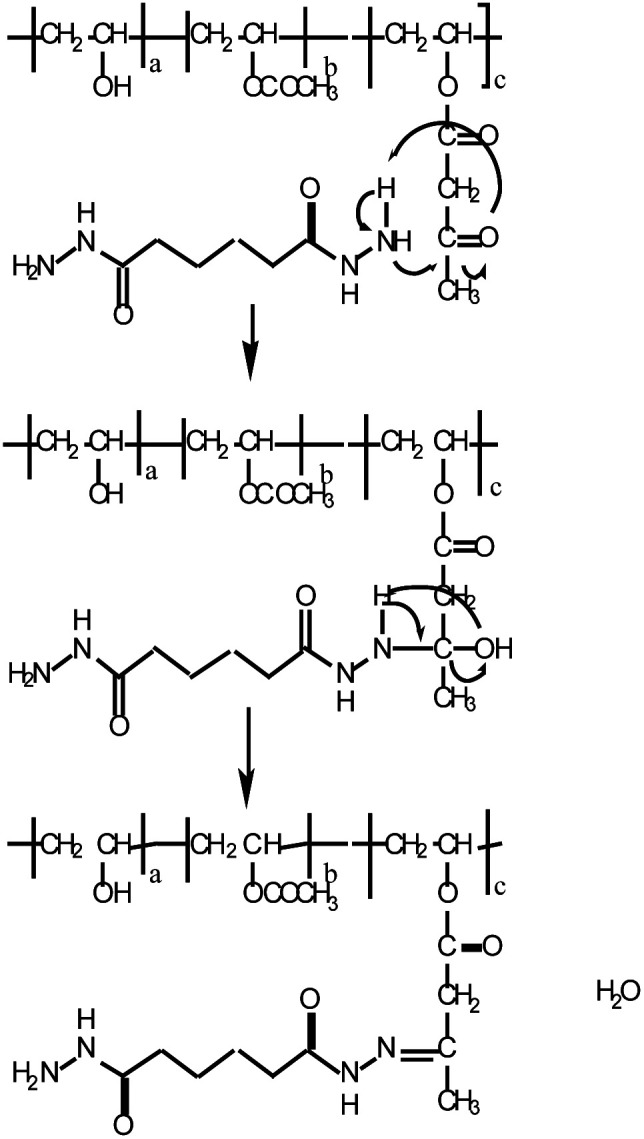
Reaction mechanism of methyl acetoacetate and acetohydrazide.

Fig. 9 shows the reaction mechanism of the reaction between methyl acetoacetate and acetohydrazide, where cross-linking takes place *via* imine bond formation.

**Fig. 10:**
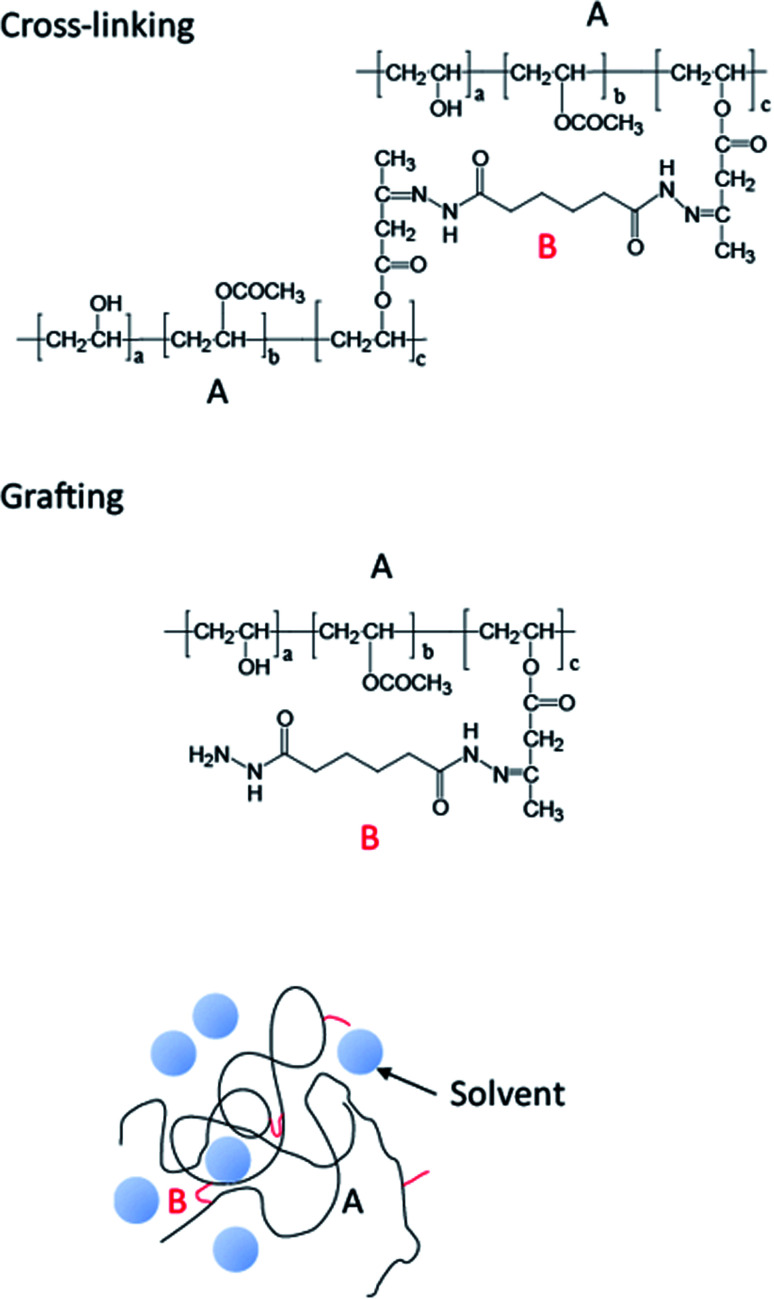
Structure of PVOH, containing a reactive acetoacetyl group, and ADH.

Fig. 10 shows that if PVOH, containing a reactive acetoacetyl group, and ADH form an A–B–A-type structure, a cross-linked structure is formed, whereas if it forms an A–B-type structure, chain extension occurs.

Although it appears that these structures coexist, because the solvent molecules (during the formation of the cross-linked structure) cannot completely loosen the polymer matrix, the overall material should be water resistant and useful as an overcoating agent for heat-sensitive recording papers. Furthermore, the integral of the peak of the imine moiety was 0.7 mol%, relative to the PVOH main chain. That is, seven cross-linked or graft structures were formed in a molecular chain that has a polymerization degree of 1000. Thus, not all of the reaction sites are involved in the cross-linking reaction; only partial reaction took place. Nonetheless, only a small degree of cross-linking provides adequate water resistance. This quantitative information is very useful in studying the relationship between cross-linked structures and water resistance.

## Conclusions

The PVOH compound was measured in the crosslinked products of PVA and ADH using a high-field DNP-NMR system to test for any improvement in sensitivity. The maximum sensitivity of *ε*_DNP_ was improved by up to 4 times, and the effect of DNP on the vinyl alcohol polymer was confirmed. Using high resolution and a high field, not only the denatured part, but also the structure of the bridging part of the compound could be detected. It was found that the carbonyl of the acetoacetyl group on PVOH reacted with the amine on ADH to form an imine (C=N) bond.

This reaction mechanism was found to be the same as the reaction between low molecular compounds (methyl acetoacetate and acetohydrazide) containing the same reactive components.

High-field DNP-NMR with high sensitivity and high resolution is a very useful method to use as a means to capture the cross-linked structure and microstructure of macromolecules, and it is expected to be used in this field in the future.

## Conflicts of interest

There are no conflicts to declare.

## Supplementary Material

RA-010-D0RA00399A-s001
