# Supplementary material for: Structural analysis of cross-linked poly(vinyl alcohol) using high-field DNP-NMR
Source: RSC Adv. 2020 Feb 24;10(14):8039–43. doi: 10.1039/d0ra00399a (PMC9049923; doi:10.1039/d0ra00399a)
Supplement: RA-010-D0RA00399A-s001 [file RA-010-D0RA00399A-s001.pdf]

Electronic Supplementary Material (ESI) for RSC Advances.
This journal is © The Royal Society of Chemistry 2020

Electronic Supplementary Information

Structural analysis of cross-linked poly(vinyl alcohol) using high-field DNP-NMR

Taiji Kanda,[*a,b] Mayuka Kitawaki,[a] Toshiaki Arata, [b] Yoh Matsuki [b], and Toshimichi Fujiwara [b]

[a] *Mitsubishi Chemical Corporation*

2-13-1, Muroyama, Ibaraki, Osaka 567-0052, Japan

[b] *Institute for Protein Research, Osaka University,*

3-2, Yamadaoka, Suita, Osaka 565-0871, Japan

Confirmation of reactive sites in the reaction between low molecular weight compounds, including acetoacetyl and hydrazide functional groups.

(The reaction of methyl acetoacetate and acetohydrazide)

Methyl acetoacetate was purchased from Fujifilm Wako Chemicals (Osaka, Japan) and Acetohydrazide was purchased from Tokyo Chemical Industry CO. Ltd. (Tokyo, Japan). These materials were used without further purification. Each material is shown in Fig. SI-1. A solution of 3.1 mg methyl acetoacetate (27 mmol), 2.0 mg acetohydrazide (27 mmol), and 40 ml of water was prepared at 23 °C. After stirring for 1 min, the solution was transferred to an NMR sample tube (the tube comprised a double tube). D_2O was placed in the outer sample tube (ϕ 5 mm) while the reaction mixture was placed in the inner sample tube (ϕ 4 mm). After mixing for 1 hour, the structure was confirmed using solution NMR, according to the conditions tabulated in Table SI-1. The solution ^{13}C NMR spectrum of the product was shown in Fig.7.

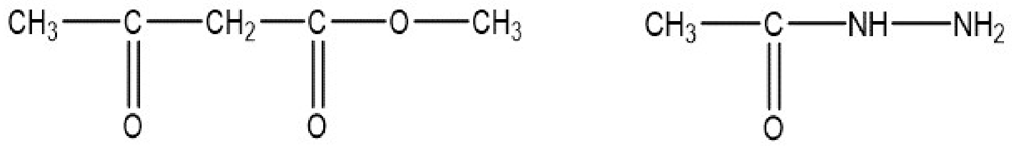

Fig. SI-1 Molecular structure of methyl acetoacetate (left) and acetohydrazide (right)

Table SI-1 Measurement conditions used for solution NMR

NMR	VARIAN UNITY-300	
	Probe	5 mm ϕ, four-nucleus (^{1}H,^{13}C,^{19}F,^{31}P) probe
	Pulse sequence	Inverse gated decoupling
	Temperature	296 K
	Spinning rate	20 Hz
	Observe nucleus	^{13}C
	90°degree pulse width	10 μs
	Decoupling nucleus	^{1}H
	Decoupling sequence	Waltz16
	Number of acquisitions	1024
